# How many neural oscillators we need on sub- and supra-second intervals processing in the primate brain

**DOI:** 10.3389/fpsyg.2014.01263

**Published:** 2014-11-06

**Authors:** Lihan Chen

**Affiliations:** Department of Psychology and Key Laboratory of Machine Perception (Ministry of Education), Peking UniversityBeijing, China

**Keywords:** interval timing, neural oscillators, models, primates, temporal processing

Processing of time intervals in short temporal ranges, typically in sub- and supra-second intervals, is vital for the survival of primates and humans. Research evidence has already shown that the processing of sub- and supra-second intervals is subserved by different neural mechanisms (Buhusi and Meck, 2005). In the domain of time perception, one outstanding scientific question remains to be solved is whether the interval processing is implemented by a central clock (supra-sensory) or specific clock (neural state-specific) representation (Ivry and Schlerf, 2008). Gupta (2014) initiated theoretical explorations by introducing different types of neuronal oscillators, with three key components: pacemaker neurons, tonic inputs, and synchronized excitation/inhibition of inter-connected neurons (Gupta, 2014). Gupta further featured three characteristic factors in an integrated model: (1). The encoding of the time interval is reflected by frequency modulated neuronal spikes or spike bursts affiliated with the temporal points of a given interval. (2). Our brain embraces active calibration mechanisms among multiple oscillators and modular connections, which could to a large extent pit against the loss of interval timing functions of the brain. (3). The neuronal oscillators have solid foundations in neural substrates that typically characterize sensorimotor timing, from the changes of membrane potential to the functioning of high-level brain structures (Teki et al., 2011; Santos et al., 2014).

The concept of the building block for “neural oscillators” in Gupta (2014) in essence could be extended and interpreted in different contexts: Firstly, the “neuronal temporal units” in Gupta (2014), in contrast to the traditional “pacemaker-accumulator” model (Treisman, 1963; Treisman et al., 1990, 1994; Wittmann, 1999), is a machinery device (inherent in the brain) that processes the time information. Secondly, the “oscillators” entail the “modular” nature of neuronal clocks, by emphasizing the correspondence between neural substrates in temporal bindings between different sensory events. In this way and from a broad perspective, this model dovetails the tenets of “intrinsic model” (Ivry and Schlerf, 2008), but allows mechanistic flexibility for the interactions between separate clocks. Thirdly, the oscillators could demonstrate higher-level ensemble neuronal activities (but were relatively less addressed in current model), including oscillatory gamma-band responses (Herrmann et al., 2004; Tallon-Baudry and Bertrand, 1999).

Movement is the expressed time (Teki et al., 2011). The Theoretical model for Gupta (2014) stems from the common setting of “movement control” but encourages more potential investigations to come. For example, it could be extended and validated in other timing scenarios—go beyond the area of motor control—to demonstrate its robustness. Moreover, a typical function of human timing system is to predict the imminent events and hence make efficient perceptual decision making. The current oscillation model leaves much room to be improved by revealing how the oscillators could work directly in a framework of predictive coding. This predictive coding is somehow free of feedback processes as assumed in Gupta (2014). Furthermore, a high-level processing of rhythm/periodicity-based upon the information of sub- or supra-second intervals, might mobilize population/ensemble neuronal coding as well as impose more complex inter-connections/overlappings of the supposed neuronal temporal units (with multiple neuronal feedback loops). What Gupta (2014) proposed essentially indicated a potential panorama of neural oscillators that could address the complexity of timing behavior. However, how many (levels of) oscillators are needed to elucidate those timing behaviors and how local lateral excitatory connectivity (Gavornik and Shouval, 2011), interacting with the global (stochastic) processing of beat timing, warrants further explorations.

## Conflict of interest statement

The author declares that the research was conducted in the absence of any commercial or financial relationships that could be construed as a potential conflict of interest.
